# Methylcrotonoyl-CoA carboxylase 1 potentiates RLR-induced NF-κB signaling by targeting MAVS complex

**DOI:** 10.1038/srep33557

**Published:** 2016-09-15

**Authors:** Zhongying Cao, Zhangchuan Xia, Yaqin Zhou, Xiaodan Yang, Hua Hao, Nanfang Peng, Shi Liu, Ying Zhu

**Affiliations:** 1 State Key Laboratory of Virology and College of Life Sciences, Wuhan University, Wuhan 430072, China

## Abstract

RNA virus infections are detected by the RIG-I family of receptors, which signal through the adaptor molecule mitochondrial antiviral signaling (MAVS). MAVS then recruits the adaptor’s tumor necrosis factor receptor-associated factor (TRAF) 3 and TRAF6, which in turn activate IRF3 and NF-κB, respectively, to induce interferons (IFNs) and inflammatory responses. Here we show that the biotin-containing enzyme methylcrotonoyl-CoA carboxylase 1 (MCCC1) enhances virus-induced, MAVS-mediated IFN and inflammatory cytokine expression through the NF-κB signaling pathway. MCCC1 knockdown strongly inhibits induction of IFNs and inflammatory cytokines. Furthermore, MCCC1 shows extensive antiviral activity toward RNA viruses, including influenza A virus, human enterovirus 71, and vesicular stomatitis virus. Here, we have elucidated the mechanism underlying MCCC1-mediated inhibition of viral replication. MCCC1 interacts with MAVS and components of the MAVS signalosome and contributes to enhanced production of type I IFNs and pro-inflammatory cytokines by promoting phosphorylation of the IκB kinase (IKK) complex and NF-κB inhibitor-α (IκBα), as well as NF-κB nuclear translocation. This process leads to activation of IFNs and cytokine expression and subsequent activation of IFN-stimulated genes, including double-stranded RNA-dependent protein kinase PKR and myxovirus resistance protein 1. These findings demonstrate that MCCC1 plays an essential role in virus-triggered, MAVS-mediated activation of NF-κB signaling.

The innate immune response is the first line of defense against pathogen invasion and is initiated upon host recognition of pathogen-associated molecular patterns by pattern recognition receptors (PRRs). Such recognition initiates signaling cascades that activate intracellular innate immune defenses and inflammatory response leading to induction of hundreds of cytokines that are involved in the repression of viral replication and clearance of infected cells^1^.

Various PRRs, which sense pathogen invasion, such as Toll-like receptors (TLRs) and retinoic acid-inducible gene I (RIG-I)-like receptors (RLRs), have been identified to date. TLRs, which are found on the cell surface or within endosomal compartments in most cells of the body, contain extracellular leucine-rich repeat motifs to recognize specific pathogens and a cytoplasmic Toll–interleukin (IL)-1 receptor domain to transmit signals^2^. TLR3 only recognizes extracellular double-stranded RNA or single-stranded RNA associated with viral particles that are internalized into the endosomes^3^,^4^. The RLRs include the cytosolic PRRs RIG-I, melanoma differentiation-associated gene 5 (MDA5), and laboratory of genetics and physiology 2 (LGP2). All three RLRs share a common structure, which includes an RNA helicase activity domain in the central portion of the protein that also contains the DExD/H box ATPase domain, a C-terminal repressor domain that is involved in autorepression of RIG-I activity, and two caspase activation and recruitment domains (CARDs) at the N-terminus, which LGP2 lacks^5^,^6^. When RIG-I senses viral RNA, it undergoes conformational changes and translocates to the mitochondria, where it interacts with the adaptor protein mitochondrial antiviral signaling (MAVS; also known as VISA, IPS-1, and Cardif)^7^,^8^,^9^,^10^. The association of RIG-I and MAVS initiates recruitment of multiple proteins to form a signalosome, leading to the bifurcation of signaling mediated either by TRAF3 to activate the type I interferons (IFNs) or by TRAF2 and TRAF6, resulting in the inflammatory response^11^. MAVS regulation of type I IFN induction is initiated by recruitment of TRAF3, which then forms a scaffold for the assembly of a signaling complex of NEMO (IKKγ) and TRAF-family member associated NF-κB activator (TANK). This complex subsequently activates TBK1 and IKKε, which specifically phosphorylate transcription factors IFN regulatory factor (IRF) 3 and IRF7, leading to their dimerization, nuclear translocation, and expression of type I IFN genes^12^,^13^. TRAF2 and TRAF6, in cooperation with receptor interacting protein 1 (RIP1) and tumor necrosis factor receptor type 1-associated death domain protein (TRADD), activates the IKK complex, consisting of IKKα, IKKβ, and IKKγ, which leads to the phosphorylation and ubiquitination of IkBa, resulting in the nuclear translocation of NF-kB and subsequent inflammatory cytokine expression^12^. Both IRFs and NF-κB bind to the IFNβ promoter in a temporally coordinated fashion to drive transcription. Secreted type I IFNs bind to and activate the type I IFN receptors to initiate the JAK/STAT pathway and transcriptional induction of a wide range of IFN-stimulated genes (ISGs). The induced downstream gene products, such as double-stranded RNA-dependent protein kinase (PKR), myxovirus resistance protein 1 (Mx1), and 2′, 5′-oligoadenylate synthetase (OAS), orchestrate the inhibition of viral replication and clearance of virus-infected cells that lead to antiviral responses^14^,^15^,^16^.

Structurally, MAVS is composed of an N-terminal CARD that interacts with RIG-I, a proline-rich domain that facilitates protein-protein interactions, and a C-terminal transmembrane domain that mediates MAVS binding to the outer mitochondrial membrane^7^,^8^,^9^,^10^,^17^. MAVS plays a central role in regulating the complex events that lead to either antiviral or inflammatory responses. Many reports have described its role in innate immune signaling. A recent study reported that phosphorylation of MAVS at multiple sites, including Ser^442^, is essential for IRF3 binding and activation^18^. Hepatitis A, B, and C viruses can interact with MAVS and block the intracellular signaling pathway at the level of MAVS^19^. An autoinhibitory mechanism that modulates MAVS activity in unstimulated cells, thus ensuring a prompt response of MAVS to virus infection, has been also reported^20^.

Methylcrotonoyl-CoA carboxylase 1 (MCCC1), located in mitochondria, is a member of the biotin-containing family of enzymes that catalyze the conversion of 3-methylcrotonyl-CoA to 3-methylglutaconyl-CoA^21^,^22^,^23^. MCCC1 plays an essential role in the catabolism of leucine and isovalerate in most living systems^24^. However, its role in innate immunity against viral infection has never been established. In this study, we demonstrate that MCCC1 inhibits the replication of RNA viruses, such as influenza A virus (IAV), vesicular stomatitis virus (VSV), and human enterovirus 71 (EV71), by targeting the MAVS signalosome to activate NF-κB signaling. These findings describe a previously unrecognized function of MCCC1.

## Results

### MCCC1 potentiates virus-triggered induction of IFN and ISG

**Figure 1:**
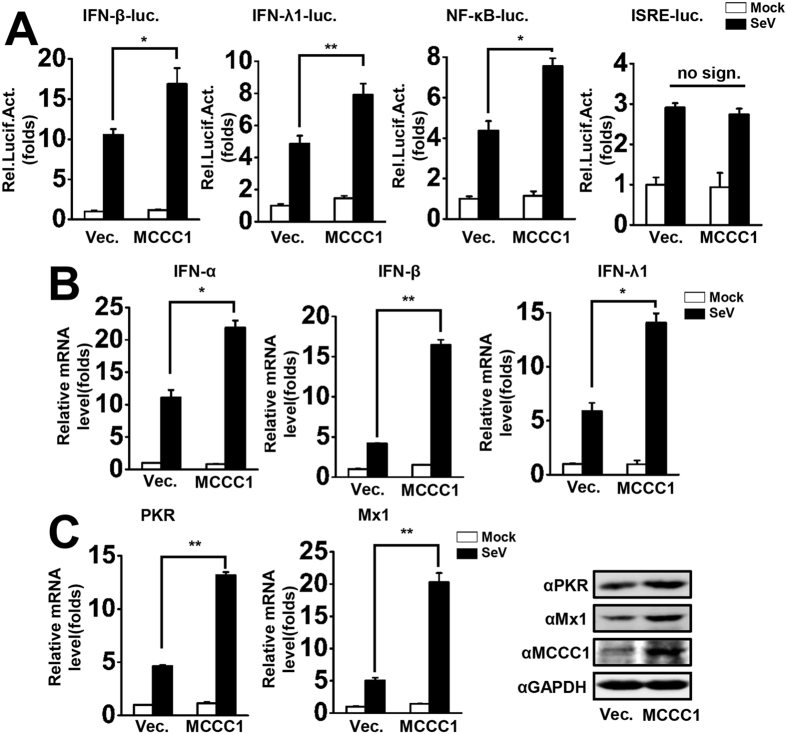
MCCC1 potentiates virus-triggered IFN and ISG induction. (**A**) A549 cells were co-transfected with IFN-β, IFN-λ1, NF-κB, and ISRE luciferase promoter constructs and pRL-TK (internal control) together with control vector or MCCC1 expression plasmid. Thirty-six hours after transfection, cells were left uninfected or infected with SeV (MOI = 1) for 12 h before luciferase assays were performed. *p < 0.05, **p < 0.01. no sign., no significant difference (one-way ANOVA). (**B**) A549 cells were transfected with vector or MCCC1 expression plasmid. Thirty-six hours after transfection, cells were left uninfected or infected with SeV (MOI = 1) for 6 h before the levels of IFNα, IFN-β, and IFN-λ1 mRNA were detected by qPCR. *p < 0.05, **p < 0.01. (one-way ANOVA). (**C**) A549 cells were transfected with vector or MCCC1 expression plasmid. Thirty-six hours after transfection, cells were left uninfected or infected with SeV (MOI = 1) for 12 h. And then, PKR and Mx1 mRNA and protein levels were assessed by qPCR and western blotting, respectively. **p < 0.01 (one-way ANOVA). All graphs represent means standard deviations for 3 experiments.

To assess whether MCCC1 is involved in the virus-triggered innate immunity signaling pathway, we performed reporter assays in A549 cells which were left uninfected or infected with SeV (MOI = 1). We found that overexpression of MCCC1 enhanced SeV-triggered activation of the IFN-β and IFN-λ1 promoters (Fig. 1A). Because IFN promoter activation requires activation of upstream components IRF3 or NF-κB, we further determined whether MCCC1 potentiated virus-induced activation of IFN-stimulated response element (ISRE) or NF-κB. The results indicated that overexpression of MCCC1 enhanced SeV-triggered activation of the NF-κB, but not the ISRE promoter (Fig. 1A), indicating that MCCC1 is only involved in activating NF-κB signaling. To assess the effect of MCCC1 on IFN mRNA expression level, real-time PCR (qPCR) was performed. The results indicated that MCCC1 overexpression potentiates SeV-triggered elevated levels of IFN-α, IFN-β, and IFN-λ1 mRNA expression (Fig. 1B). Because IFNs activation induces the transcription of hundreds of ISGs, we determined the mRNA and protein levels of ISGs including PKR and Mx1 by qPCR and western blotting, respectively. The results showed that MCCC1 overexpression significantly increased the levels of PKR and Mx1 mRNA and protein (Fig. 1C). Taking these data together, we concluded that MCCC1 potentiates virus-triggered induction of IFNs and ISGs.

### Knockdown of MCCC1 inhibits IFN and ISG activation

**Figure 2:**
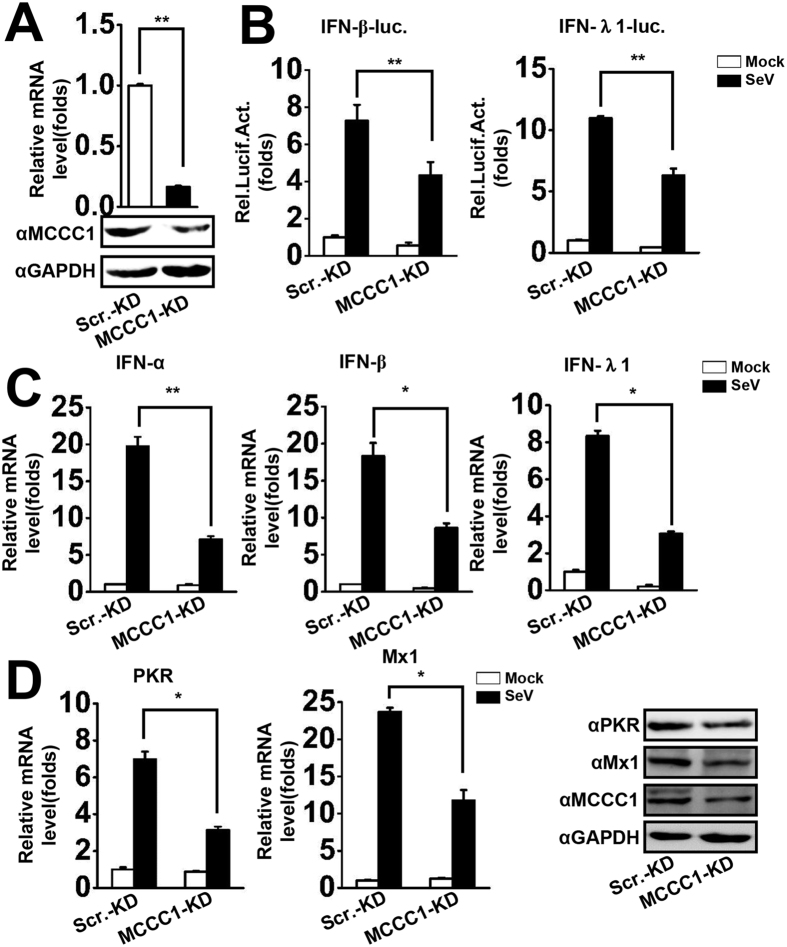
Knockdown of MCCC1 inhibits IFN and ISG activation. (**A**) A549 MCCC1-KD and Scr.-KD cells were collected to assess MCCC1 mRNA and protein levels by qPCR and western blotting, respectively. **p < 0.01 (one-way ANOVA). (**B**) IFN-β and IFN-λ1 luciferase promoter constructs and pRL-TK (internal control) were co-transfected into MCCC1-KD or Scr.-KD cells. Thirty-six hours after transfection, cells were left uninfected or infected with SeV (MOI = 1) for 12 h before luciferase assays were performed. **p < 0.01 (one-way ANOVA). (**C**) The same numbers of MCCC1-KD and Scr.-KD cells were left uninfected or infected with SeV (MOI = 1) for 6 h. IFN mRNA levels were measured by qPCR. *p < 0.05, **p < 0.01 (one-way ANOVA). (**D**) The same numbers of MCCC1-KD and Scr.-KD cells were left uninfected or infected with SeV (MOI = 1) for 12 h. PKR and Mx1 mRNA and protein levels were determined by qPCR and western blotting, respectively. *p < 0.05. (one-way ANOVA). All graphs represent means standard deviations for 3 experiments.

Because MCCC1 overexpression upregulated SeV-triggered induction of IFNs and ISGs, we next determined whether endogenous MCCC1 is required for virus-triggered IFN induction. We generated a stable MCCC1 knockdown A549 cell line using a vector-based shRNA in a lentiviral system^25^. MCCC1 was significantly reduced at both the mRNA and protein levels in cells transduced with recombinant lentivirus containing shRNA targeting MCCC1 (MCCC1-KD) compared to those infected with recombinant lentivirus containing scrambled control shRNA (Scr.-KD) (Fig. 2A). Results of reporter assays indicated that MCCC1 knockdown decreased SeV-triggered IFN-β and IFN-λ1 promoter induction (Fig. 2B). IFN mRNA levels were also assessed by qPCR. The data showed that MCCC1 knockdown repressed virus-triggered IFN mRNA expression (Fig. 2C). Subsequently, we examined the effect of MCCC1 knockdown on virus-induced expression of ISGs including PKR and Mx1 by qPCR and western blot analyses in MCCC1-KD cells. The MCCC1-KD cells showed lower levels of PKR and Mx1 mRNA and protein than Scr.-KD cells (Fig. 2D). Further, we synthesized three short interfering (si) RNAs targeting MCCC1 (siMCCC1- #1–3), and the effect of the siRNAs on MCCC1 expression in A549 cells was determined by qPCR. The results demonstrated that all three siRNAs effectively knocked down MCCC1 expression (Supplementary Figure S1A). Because MCCC1 is a biotin-containing enzyme including in metabolism, the MTS assay was performed to assess the effect of MCCC1 depletion on cell viability. We found that transfection of cells with siMCCC1 - #1, #2, or #3 had no significant impact on viability of A549 cells (Supplementary Figure S1B). Because siMCCC1- #3 was the most efficient in knocking down MCCC1, we used siMCCC1- #3 in subsequent experiments. Results of reporter assays indicated that MCCC1 knockdown inhibited virus-triggered activation of IFN-β and IFN-λ1 promoter activity (Supplementary Figure 1C). Collectively, these results demonstrate that MCCC1 is required for virus-triggered induction of IFNs and ISGs.

### MCCC1 is a positive regulator of virus-induced pro-inflammatory cytokines

**Figure 3:**
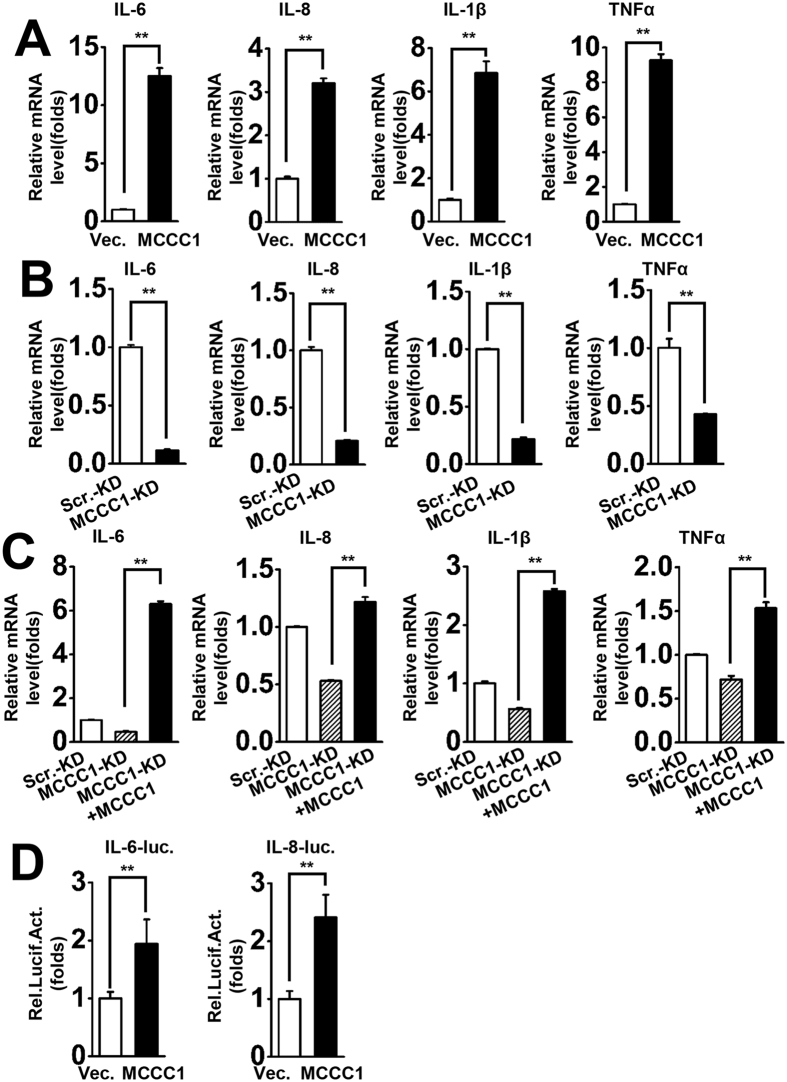
MCCC1 is a positive regulator of virus-induced pro-inflammatory cytokines. (**A**) The vector or MCCC1 expression plasmids were transfected to A549 cells for 36 h and then infected with SeV (MOI = 1) for 6 h. The mRNA levels of IL-6, IL-8, IL-1β, and TNFα were measured by qPCR. (**B**) The same numbers of MCCC1-KD and Scr.-KD cells were infected with SeV (MOI = 1) for 6 h. The mRNA levels of IL-6, IL-8, IL-1β, and TNFα were measured by qPCR. (**C**) MCCC1-KD cells and Scr.-KD cells were transfected with vector or MCCC1 expression vector for 36 h and then infected with SeV. Levels of IL-6, IL-8, IL-1β, and TNFα mRNA were measured by qPCR. (**D**) A549 cells were co-transfected with IL-6 or IL-8 luciferase reporter plasmids and pRL-TK (internal control) together with vector or MCCC1 expression vector. Thirty-six hours after transfection, cells were infected with SeV for 12 h before reporter assays were performed. **p < 0.01 (one-way ANOVA). All graphs represent means standard deviations for 3 experiments.

As our results showed that overexpression of MCCC1 enhanced SeV-triggered activation of the NF-κB promoter, we further examined the expression of NF-κB–responsive genes encoding pro-inflammatory cytokines, including IL-6, IL-8, IL-1β, and TNFα^26^. The results of qPCR analysis demonstrated that overexpression of MCCC1 greatly increased virus-induced expression of these pro-inflammatory cytokines at the mRNA level (Fig. 3A). Subsequently, we assessed the role of endogenous MCCC1 on expression of virus-triggered pro-inflammatory cytokines. We assessed levels of IL-6, IL-8, IL-1β, and TNFα mRNA in MCCC1-KD cells infected with SeV. The results showed that MCCC1-KD cells expressed much lower levels of cytokine mRNA than Scr.-KD cells upon virus infection (Fig. 3B). We next restored MCCC1 production by transfecting MCCC1-KD cells with MCCC1 expression plasmid, and assessed the expression of IL-6, IL-8, IL-1β, and TNFα at the mRNA level by qPCR. Interestingly, restoration of MCCC1 expression in MCCC1-KD cells rescued the downregulation of IL-6, IL-8, IL-1β, and TNFα (Fig. 3C). This result indicated that MCCC1 depletion in MCCC1-KD cells was responsible for the downregulation of pro-inflammatory cytokines. Subsequently, we performed reporter assays, and these showed that overexpression of MCCC1 enhanced virus-triggered activation of the IL-6 and IL-8 promoters (Fig. 3D). Taken together, these data indicate that MCCC1 enhances virus-induced expression of pro-inflammatory cytokines.

### MCCC1 is essential for cellular antiviral responses

**Figure 4:**
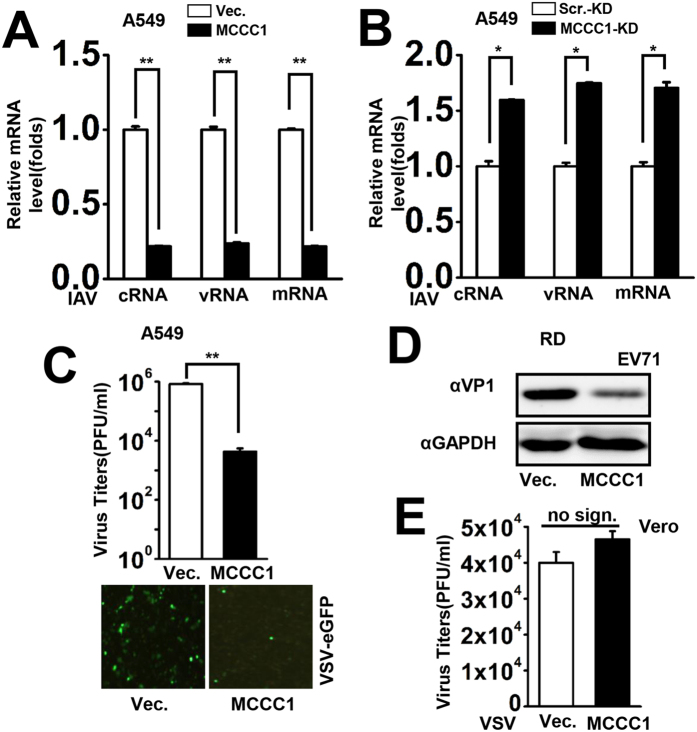
MCCC1 is essential for cellular antiviral responses. (**A**) A549 cells were transfected with vector or MCCC1 expression plasmids for 24 h before IAV infection (MOI = 1). Cells were collected 24 h post-infection and total RNA was isolated for detection of NP-specific mRNA, cRNA and vRNA. **p < 0.01 (one-way ANOVA). (**B**) The same numbers of MCCC1-KD and Scr.-KD cells were infected with IAV (MOI = 1) for 24 h. The NP-specific mRNA, cRNA and vRNA were assessed by qPCR. *p < 0.05 (one-way ANOVA). (**C**) A549 cells were transfected with vector or MCCC1 expression vector for 24 h and then infected with VSV-eGFP (MOI = 1). Twenty-four hours post-infection, the supernatants were harvested and analyzed for VSV production using a standard plaque assay (upper panel). **p < 0.01 (one-way ANOVA). After infection for 6 h, VSV-eGFP replication was visualized by fluorescence microscopy (lower panel). (**D**) RD cells were transfected with vector or MCCC1 expression vector for 24 h. Twelve hours after EV71 infection (MOI = 1), VP1 levels were measured by western blotting. (**E**) Vero cells were transfected with vector or MCCC1 expression vector for 24 h and then infected with VSV (MOI = 1) for another 24 h. VSV in supernatants was assessed by a standard plaque assay. no sign., no significant difference. (one-way ANOVA). All graphs represent means standard deviations for 3 experiments.

Having demonstrated that MCCC1 is a positive regulator of virus-induced IFN and pro-inflammatory cytokine expression, we speculated that MCCC1 might be involved in host antiviral activity. To explore this possibility, A549 cells overexpressing MCCC1 were infected with viruses IAV, EV71, and VSV. The effect of MCCC1 overexpression on IAV nucleoprotein (NP) cRNA, vRNA, and mRNA levels was determined by qPCR. Significantly, MCCC1 inhibited the expression of all NP gene mRNAs (Fig. 4A). To further confirm the role of endogenous MCCC1 in antiviral activity, we assessed IAV replication in MCCC1-KD cells and found that depletion of MCCC1 increased IAV NP gene expression, including cRNA, vRNA, and mRNA (Fig. 4B). We then detected the effect of MCCC1 overexpression in A549 cells on recombinant VSV-enhanced green fluorescent protein (eGFP) replication. In plaque assays, we observed lower viral titers in MCCC1-overexpressing cells than in control cells (Fig. 4C, top). Moreover, VSV-eGFP replication was visualized by fluorescence microscopy, and it was clear that MCCC1 expression inhibited VSV-eGFP expression (Fig. 4C, bottom). Next, RD cells were transfected with MCCC1 expression vector or vector alone and infected with EV71 for 12 h. VP1, a capsid protein of EV71, was detected by western blotting, which indicated that MCCC1 expression repressed EV71 expression in RD cells (Fig. 4D). Thus, we showed that MCCC1 has antiviral activity toward several RNA viruses, IAV, VSV, and EV71. To determine whether the antiviral activity of MCCC1 depends on the presence of IFN, we transfected Vero cells, a cell line lacking functional type I IFN genes^27^,^28^,^29^, with MCCC1 expression vector or control vector and infected the cells with VSV. We observed no significant difference in virus titer in MCCC1-overexpressing and control cells, indicating that MCCC1 has no anti-VSV activity in Vero cells (Fig. 4E) and that MCCC1 exerts its antiviral activity through the IFN signaling pathway.

### MCCC1 targets the MAVS complex

**Figure 5:**
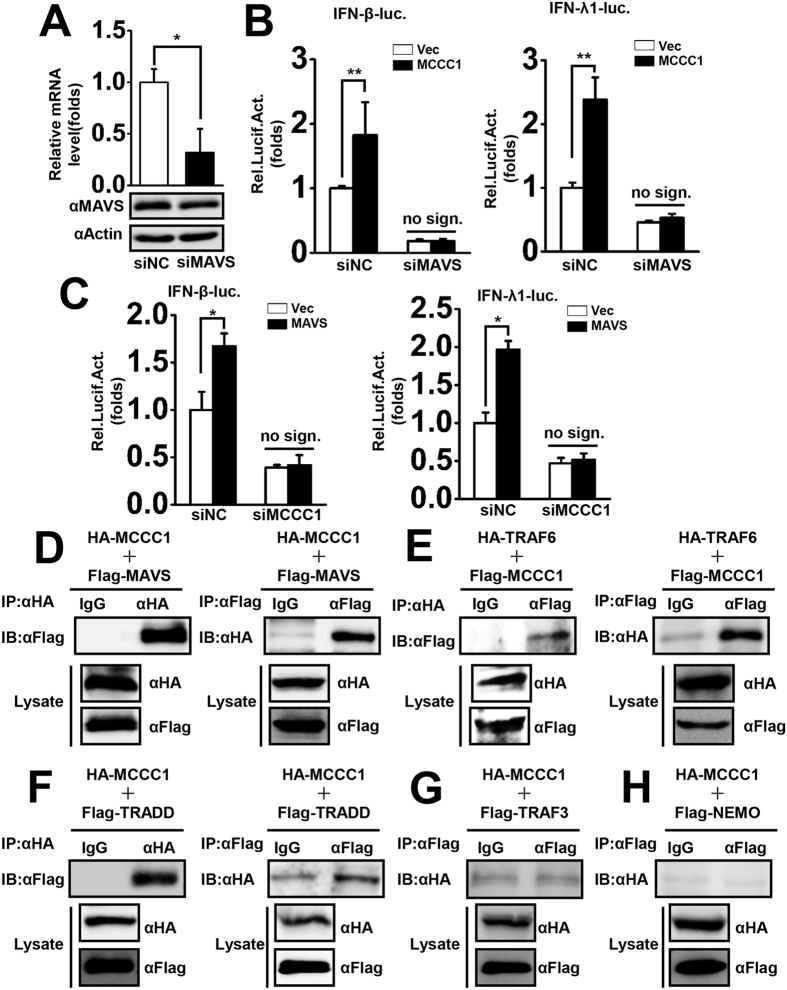
MCCC1 targets MAVS complex. (**A**) A549 cells were transfected with siRNA targeting MAVS or nonsense control siRNA for 48 h, and MAVS mRNA and protein levels were measured by qPCR and western blotting, respectively. *p < 0.05 (one-way ANOVA). (**B**) A549 cells were co-transfected with vector alone or MCCC1 expression vector and the indicated siRNA together with IFN-β and IFN-λ1 luciferase reporter plasmids and pRL-TK (internal control). Twenty-four hours after transfection, cells were infected with SeV (MOI = 1) for 12 h before reporter assays were performed. **p < 0.01, no sign., no significant difference. (one-way ANOVA). (**C**) A549 cells were co-transfected with control vector alone or MAVS expression plasmid and MCCC1-specific siRNA together with IFN-β and IFN-λ1 luciferase reporter plasmids and pRL-TK (internal control). Twenty-four hours after transfection, the cells were infected with SeV (MOI = 1) for 12 h before reporter assays were performed. **p < 0.01, no sign., no significant difference. (one-way ANOVA). (**D**–**H**) HEK293T cells were co-transfected with HA-MCCC1 and FLAG-MAVS expression vectors (**D**), HA-TRAF6 and FLAG-MCCC1 expression vectors (**E**), HA-MCCC1 and FLAG-TRADD expression vectors (**F**), HA-MCCC1 and FLAG-TRAF3 expression vectors (**G**), or HA-MCCC1 and FLAG-NEMO expression vectors (**H**) for 48 h. Co-immunoprecipitation and immunoblot analyses were performed with the indicated antibodies. All experiments were repeated at least three times with consistent results.

MCCC1 is a mitochondrial protein, and our results demonstrate that MCCC1 is involved in virus-triggered induction of IFNs and pro-inflammatory cytokines and the antiviral process. Moreover, MAVS is located on mitochondria and plays a central role in regulating the complex events that lead to either antiviral or inflammatory responses. For these reasons, we wondered whether MCCC1-mediated IFN induction and inflammatory responses are MAVS-dependent. We designed siRNA targeting MAVS and tested its knockdown efficiency in A549 cells at both the mRNA and protein levels (Fig. 5A). Subsequently, we performed reporter assays to determine the effect of MAVS depletion on MCCC1-induced IFN-β and IFN-λ1 promoter activity. The results indicated that MAVS knockdown significantly inhibited MCCC1-induced IFN promoter induction (Fig. 5B), suggesting that the MCCC1-mediated upregulation of IFNs upon virus infection is MAVS-dependent. We next detected the role of MCCC1 in MAVS signaling pathway. MCCC1 specific siRNA-#3 was used to knock down the expression of MCCC1, and we then assessed the MAVS-induced IFN-β and IFN-λ1 promoter activity. The data demonstrated that knockdown of MCCC1 abolished MAVS-induced IFN promoter induction (Fig. 5C), implying that MCCC1 plays an essential role in MAVS signaling pathway. Furthermore, we examined the relationship between MCCC1 and components of the MAVS signalosome by transient transfection of HEK293T cells with HA-MCCC1 and FLAG-MAVS expression vectors, followed by co-immunoprecipitation with anti-HA antibody and found that HA-tagged MCCC1 co-immunoprecipitated with FLAG-tagged MAVS. This finding was confirmed by reciprocal co-immunoprecipitation with anti-FLAG antibody (Fig. 5D). Similar sets of experiments showed that HA-tagged TRAF6 interacted with FLAG-tagged MCCC1 (Fig. 5E) and FLAG-tagged TRADD interacted with HA-tagged MCCC1 (Fig. 5F); however, HA-tagged MCCC1 did not interact with FLAG-tagged TRAF3 (Fig. 5G), which interacts with MAVS to activate the ISRE promoter. These results are consistent with our finding that MCCC1 activates the NF-κB, but not the ISRE, promoter. NEMO, a downstream signaling component of TRAF3, also showed no interaction with MCCC1 in co-immunoprecipitation experiments (Fig. 5H). These data show that MCCC1 targets the MAVS-TRAF6 signalosome.

### Endogenous MCCC1 interacts and colocalizes with MAVS and TRAF6

**Figure 6:**
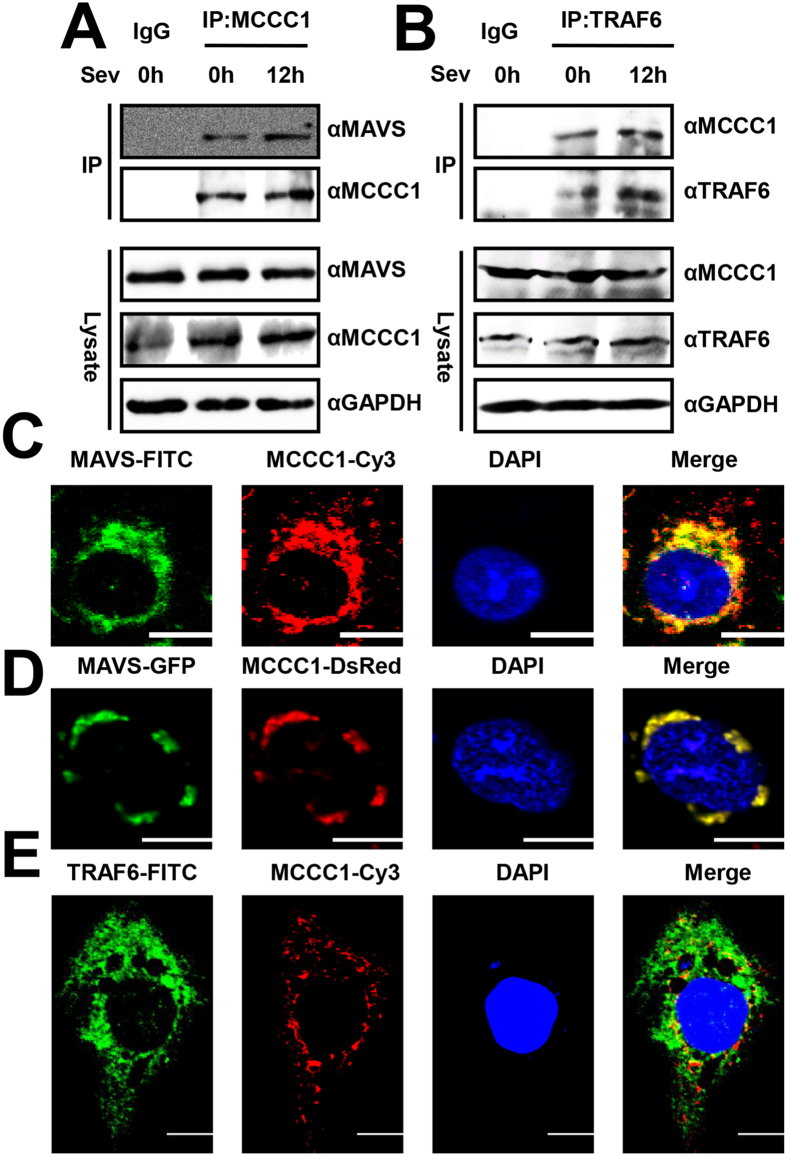
Endogenous MCCC1 interacts and colocalizes with MAVS and TRAF6. (**A**,**B**) Western analysis of results of co-immunoprecipitation of MCCC1 and MAVS (**A**) and MCCC1 and TRAF6 (**B**) from SeV-infected or uninfected A549 cells using anti-MCCC1 (**A**) and anti-TRAF6 (**B**) antibody or control IgG. (**C**,**D**) Confocal immunofluorescence microscopic analysis of MCCC1 and MAVS in A549 cells endogenous (**C**) and 24 h after transfection with DsRed-MCCC1 and GFP-MAVS expression plasmids (**D**). (**E**) Confocal immunofluorescence microscopic analysis of MCCC1 and TRAF6 in A549 cells 24 h after transfection with HA-TRAF6 expression vector. Nuclei were counterstained with DAPI. Scale bar, 10 μm. All experiments were repeated at least three times with consistent results.

Because MCCC1 is involved in RLR-mediated signaling pathways, we examined associations between endogenous MAVS, TRAF6, and MCCC1. A549 cells were infected with SeV for 12 h or left uninfected. The harvested cells were then lysed and analyzed in co-immunoprecipitation experiments. MCCC1 co-precipitated with MAVS, showing that the endogenous proteins interact (Fig. 6A). Similar experiments also revealed that MCCC1 was constitutively associated with TRAF6 (Fig. 6B). To further confirm the interactions between MCCC1 and MAVS or TRAF6, we analyzed the cells by confocal microscopy. We observed that the intracellular distribution pattern of MCCC1 was similar to MAVS (Fig. 6C). Additionally, we performed exogenous confocal assay, A549 cells were transfected with the plasmids expressing DsRed-MCCC1 and GFP-MAVS for 24 h. Similar distribution pattern between MCCC1 and MAVS was detected using fluorescence microscopy (Fig. 6D). The same results we observed between MCCC1 and TRAF6 (Fig. 6E), which is consistent with the co-immunoprecipitation results. These findings demonstrate that endogenous MCCC1 and MAVS-associated factors interact.

### Effects of MCCC1 on the phosphorylation of IκBα and nuclear translocation of NF-κB

RIG-I–like receptors sense RNA virus infection and initiate a complex signaling cascade by recruitment of MAVS to orchestrate the innate host response to virus. This process ultimately leads to the induction of antiviral and inflammatory responses mediated by the type I IFN and NF-κB pathways^30^. Because our results showed that MCCC1 is involved in virus-triggered induction of IFNs and pro-inflammatory cytokines, and MCCC1 is involved in activation of NF-κB but not ISRE signaling.

**Figure 7:**
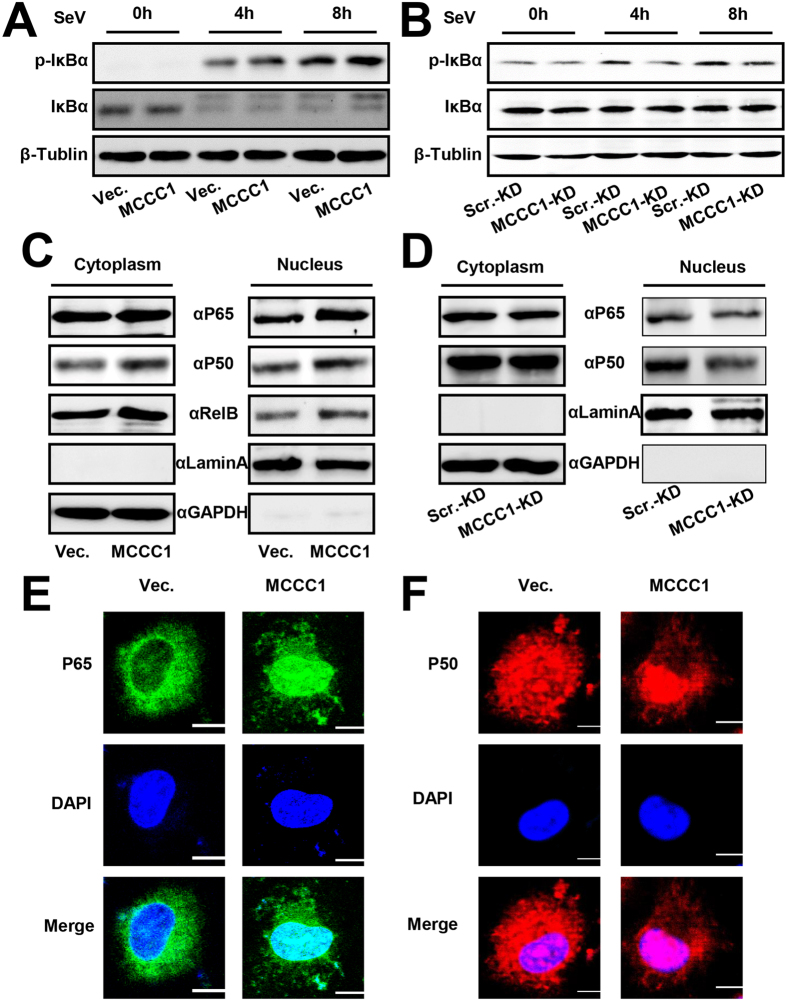
Effect of MCCC1 on phosphorylation of IκBα and nuclear translocation of NF-κB. (**A**–**D**) Western analysis of the indicated proteins, (**A**) A549 cells transfected with vector alone or MCCC1 expression vector for 36 h and then infected with SeV for the indicated times; (**B**) Scr.-KD cells and MCCC1-KD cells infected with SeV for the indicated times; (**C**) cytoplasmic and nuclear extracts of A549 cells transfected with vector alone or MCCC1 expression vector for 36 h and then infected with SeV for 12 h; GAPDH and lamin A served as markers of cytoplasm and nucleus, respectively; and (**D**) cytoplasmic and nuclear extracts of Scr.-KD cells and MCCC1-KD cells infected with SeV for 12 h. (**E**,**F**) Confocal immunofluorescence microscopic analysis of P65 (**E**) and P50 (**F**) in A549 cells transfected with vector alone or MCCC1 expression vector for 48 h. Nuclei were counterstained with DAPI. Scale bar, 10 μm. Data are representative of at least three independent experiments.

The common step in the NF-kB activation process is mediated by an IκB kinase (IKK) complex that phosphorylates IκBα and targets it for ubiquitination and proteasomal degradation, thus promoting NF-κB nuclear translocation^31^,^32^. Therefore, we examined the effect of MCCC1 on IκBα phosphorylation. A549 cells were transfected with MCCC1 expression plasmid or vector alone and infected with SeV. Western blot analysis showed that MCCC1 overexpression significantly promoted IκBα phosphorylation (Fig. 7A). To determine whether MCCC1 is required for IκBα phosphorylation, we performed similar experiments in MCCC1-KD cells and found that knockdown of MCCC1 inhibited virus-triggered phosphorylation of IκBα (Fig. 7B). When IκBα, an inhibitor of NF-κB, was phosphorylated and degraded, NF-κB was activated and translocated to the nucleus. Therefore, we next assessed the effect of MCCC1 overexpression on NF-κB translocation from the cytosol to the nucleus. Western blot analyses revealed that levels of NF-κB subunits P65 and P50 were elevated in the nucleus 48 h after MCCC1 expression (Fig. 7C). Stable MCCC1-KD cells infected with SeV were used to investigate the effect of endogenous MCCC1 on NF-κB translocation. We found that in these cells virus-triggered nuclear translocation of P65 and P50 was greatly reduced (Fig. 7D). Similar results were observed by immunofluorescence analysis (Fig. 7E,F). These results are consistent with our observations that MCCC1 enhances RLR-mediated NF-κB activation and expression of IFNs and pro-inflammatory cytokines.

## Discussion

The mitochondrial enzyme MCCC1 belongs to the family of biotin-dependent carboxylases and catalyzes the fourth step in the leucine catabolic pathway^33^. MCCC1 harbors the biotin carboxylase domain and the biotin carboxyl carrier-protein domain covalently bound to a biotin prosthetic group^34^. Many reports have described MCCC1 activity deficiencies in humans, which are linked to 3-methylcrotonylglycinuria (MCG). MCG constitutes one of the most frequently observed inborn errors of metabolism, and its clinical manifestations are highly variable, ranging from asymptomatic individuals to neonatal onset with severe cases resulting in death^22^,^35^,^36^,^37^,^38^,^39^,^40^,^41^. However, MCCC1 involvement in the innate immune system has never been reported. In this study, we demonstrated that MCCC1 is a positive regulator of virus-triggered IFN and pro-inflammatory cytokine expression through targeting of MAVS and components of the MAVS signalosome and also exhibits strong antiviral activity toward RNA viruses. In recent studies, more and more new regulators of MAVS have been reported. For example, mitochondria-associated endoplasmic reticulum membrane protein Gp78 regulates MAVS signaling by altering MAVS expression and degradation^42^. UBXN1 potently inhibits RLR- and MAVS-induced innate immune responses by targeting MAVS and disrupting the MAVS/TRAF3/TRAF6 signalosome^43^. PCBP2 mediated MAVS degradation to inhibit MAVS induced signaling pathway^44^.

Overexpression of MCCC1 potentiated virus-triggered activation of the IFN and pro-inflammatory cytokine promoters and induction of the endogenous IFN and pro-inflammatory cytokine genes, whereas MCCC1 knockdown had the opposite effect, suggesting that MCCC1 is an important component of innate immune signaling pathways in response to viral infection. Thus, MCCC1 exhibits strong antiviral activity toward RNA viruses, such as IAV, EV71, and VSV, which are potent agonists of the RLR signaling pathway^17^,^45^,^46^. Moreover, knockdown of MAVS significantly repressed MCCC1-mediated increase in virus-triggered IFNβ and IFNλ1 promoter activation. And the knockdown of MCCC1 can also suppress MAVS signaling transduction. MAVS is key adapter molecule in the RLR signaling pathway^10^, and MCCC1 interacted with MAVS and MAVS-associated factors TRAF6 and TRADD. Taking these data together, we conclude that MCCC1 is involved in the virus-triggered RLR signaling pathway to enhance induction of IFN and pro-inflammatory cytokine expression and repress RNA virus replication. Many studies have shown that mitochondrial proteins facilitate MAVS signaling pathways to inhibit viral replication. Evolutionarily conserved signaling intermediate in Toll pathway (ECSIT) localizes to mitochondria through its N-terminal domain and exerts an antiviral effect by directly interacting with RIG-I, MDA5, and MAVS, and facilitating MAVS recruitment by RIG-I^47^. Translocases of outer membrane 70 (Tom70), a mitochondrial import receptor, interacts with MAVS upon RNA viral infection to enhance IRF3-mediated gene expression and facilitate host antiviral effects^48^. MITA localizes to the outer membrane of mitochondria and associates with MAVS to activate virus-triggered IRF3 induction and interferon expression^49^,^50^. Here, we newly identify MCCC1 as a component of the MAVS signaling complex on mitochondria that enhances MAVS signaling.

We also used shRNA-mediated knockdown to generate a stable MCCC1 knockdown cell line to determine whether endogenous MCCC1 is required for virus-induced IFN and pro-inflammatory cytokine expression. In a study of the role of major vault protein (MVP) in dsRNA- or virus-induced inflammatory responses, this technique was used to knockdown MVP expression for further study^51^. The growth factor receptors erbB2, erbB3, and insulin-like growth factor-I receptor were knocked down by this technique to assess their function^25^. In this study, we also used this technique to silence MCCC1 expression in A549 cells, which provided a reliable and stable MCCC1 knockdown environment. This allows us more confidence in our observation that the absence of MCCC1 repressed virus-triggered NF-κB activation and subsequent upregulation of IFN and pro-inflammatory cytokines. We restored MCCC1 expression in MCCC1-KD cells, and showed that override of MCCC1 knockdown rescued the effect of MCCC1 on expression of pro-inflammatory cytokines IL-6, IL-8, IL-1β, and TNFα. Here we should note that when MCCC1 expression was restored in the knockdown cells, NF-κB–responsive cytokine production was greatly enhanced. This can be explained in that these inflammatory cytokines were too sensitive to MCCC1 protein mediated signaling. Another point that should be emphasized is that overexpression of MCCC1 particularly enhanced virus-triggered NF-κB, but not ISRE, promoter activation. This is in agreement with the finding that MCCC1 interacts with TRAF6, which associates with TRAF2 and RIP1 and activates the classical kinase complex-IKKα, IKKβ, and NEMO-resulting in specific activation of NF-κB but not IRFs. These findings are all sufficiently consistent to demonstrate that MCCC1 activates virus-triggered NF-κB through the MAVS-TRAF6 signaling pathway. It is well known that NF-κB is present in the cytoplasm in association with inhibitory IκB proteins. Upon viral infection, phosphorylated IKK complexes phosphorylate IκBα, which is subsequently ubiquitinated and degraded by the proteasome. NF-κB is then released and translocates to the nucleus^8^,^10^; we also assessed this process to confirm the mechanism of MCCC1 activation of NF-κB signaling. We found that MCCC1 increases the phosphorylation levels of IκBα and promotes NF-κB nuclear translocation. Moreover, MCCC1 depletion has the opposite effect. In conclusion, MCCC1 enhances virus-triggered activation of the innate immune response through RIG-I–MAVS–NF-κB signaling and induces the expression of IFNs and pro-inflammatory cytokines to hamper virus replication.

## Methods

### Plasmids and Reagents

Human MCCC1 cDNA was purchased from Proteintech Group (Rosemont, IL, USA) amplified by PCR, and cloned into one of three sites: the EcoRI/HindIII site of pCMV-tag2B to generate plasmid FLAG2B-MCCC1; the EcoRI/BamHI site of pCMV-14-3 × FLAG to generate plasmid FLAG-MCCC1; the HindIII/SalI site of PKH3-3 × HA to generate plasmid HA-MCCC1; or the EcoRI/BamHI site of DsRed to generate plasmid DsRed-MCCC1. Expression vectors for TRADD, NEMO, TRAF3, MAVS, and TRAF6 were constructed in our lab. The IFN-β, IFN-λ1, IL-6, IL-8, ISRE, and NF-κB promoter/reporter constructs were described elsewhere^52^,^53^. All constructs and gift plasmids were confirmed by DNA sequencing.

The small interfering RNA (siRNA) plasmids directed against MCCC1 and MAVS were purchased from Guangzhou RiboBio Group (Guangzhou, China). The MAVS-targeting siRNA sequence was described in previous report^54^.

Polyclonal rabbit antibodies against human MCCC1, human TRAF6, human P65, human P50, human RelB, and human laminA were purchased from Santa Cruz Biotechnology (Santa Cruz, CA, USA). Monoclonal mouse antibody against viral protein VP1 and human MAVS were also purchased from Santa Cruz Biotechnology. Polyclonal rabbit antibodies against human PKR and human Mx1 were purchased from Origene (Rockville, MD, USA). Polyclonal rabbit antibody against human IkBα and monoclonal mouse antibody against human p-IkBα were purchased from Cell Signaling Technology (Danvers, MA, USA). Monoclonal mouse antibody against HA and FLAG were purchased from MBL (Woburn, MA, USA). Monoclonal mouse antibodies against GAPDH and β-tubulin were purchased from Invitrogen (Carlsbad, CA, USA).

### Virus and Cell Culture

The influenza virus A/Hong Kong/498/97 (H3N2) strain was provided by the China Center for Type Culture Collection^55^. Recombinant VSV carrying the enhanced green fluorescent protein gene (VSV-eGFP) and the EV71 strain were described previously^56^. Human lung epithelial cells (A549) were cultured in F12K medium (Invitrogen) supplemented with 10% fetal bovine serum (FBS). 293T, Huh7, and Vero cells were cultured in Dulbecco’s modified Eagle medium (DMEM) containing 10% FBS. RD cells were cultured in RPMI 1640 medium containing 10% FBS. All cell cultures were maintained at 37 °C in a 5% CO_2_ incubator.

### Measurement of IAV replication

A549 cells were infected with IAV/Hong Kong/498/97 (H3N2) (MOI = 1). Cells were collected 24 h post-infection and total RNA was isolated. Relative levels of IAV NP viral RNA (vRNA), cRNA, and mRNA were reverse transcribed and amplified by qPCR. The following primers were used for reverse transcription: NP-vRNA, 5′-CTCACCGAGTGACATCAACATCATG-3′; NP-cRNA, 5′-AGTAGAAACAAGGGTATTTTTCTTTAATTGTCAT-3′; and NP-mRNA, oligo(dT)^56^,^57^,^58^. The following primers were used for qPCR: NP, 5′-ATCAGACCGAACGAGAATCCAGC-3′ (sense) and 5′-GGAGGCCCTCTGTTGATTAGTGT-3′ (antisense).

### Gene silencing with lentivirus encoding specific shRNA

The lentiviral pLKO.1-TRC cloning vector was a gift from Professor Yingliang Wu of Wuhan University, China. Lentivirus containing specific shRNA was produced as described in the Addgene pLKO.1-TRC cloning vector protocol. Briefly, shRNA expression plasmid encoding negative control or MCCC1 shRNA (shNC or shMCCC1, respectively), lentivirus packaging plasmid psPAX2, and envelope plasmid pMD2.G were co-transfected into 293T cells in 60-mm culture dishes with Lipofectamine 2000 (Invitrogen). After 24 h, we harvested the medium, transferred it to a polypropylene storage tube, and then added 5 mL fresh medium to the cells and incubated them for another 24 h. The harvested medium was centrifuged at 15,000 g for 5 min and filtered through a 0.45-μm filter (Millipore, Burlington, MA, USA) to remove cells. The shMCCC1 sequence, CCGGGCGAAGCTGATTATCCTGGAACTCGAGTTCCAGGATAATCAGCTTCGCTTTTTG, was obtained from the Sigma website and was validated.

Lentiviral vectors containing specific shRNAs were used to infect A549 cells in the presence of 8 μg/mL polybrene. Twenty-four hours after infection, virus-infected cells were selected by culture in puromycin (1 μg/mL) for 72 h and assessed by qPCR and western blotting to confirm MCCC1 knockdown. The resulting cell line was designated MCCC1-KD.

### Western blot analysis and co-immunoprecipitation

Whole-cell lysates were prepared by suspending cells in lysis buffer (0.01% EDTA, 0.1% Triton X-100, and 10% proteinase inhibitor mixture), followed by brief sonication, and centrifugation at 15,000 g for 15 min. The supernatants were precleared by incubation with protein G PLUS-Agarose beads (Roche, Basel, Switzerland) for 1 h followed by centrifugation at 15,000 g for 1 min. The supernatants were incubated with appropriate antibody and cross-linked to protein G PLUS-Agarose beads overnight. Beads were washed five times before the proteins were eluted by boiling for 10 min in sodium dodecyl sulfate (SDS) sample buffer.

Western blotting was performed as described previously^56^,^59^. Briefly, 30 μg of total protein or an aliquot of precipitated protein was resolved on 12% SDS-polyacrylamide gel electrophoresis (SDS-PAGE) gels and transferred onto nitrocellulose membranes (Bio-Rad, Hercules, CA, USA). Membranes were blocked with nonfat milk for 1 h at room temperature and then incubated with a primary antibodies diluted in PBS overnight at 4 °C. Secondary antibodies (1:5,000) was then added, and membranes were incubated at room temperature for 1 h. The immunoblots were visualized using a LAS-4000 image document instrument (FujiFilm, Tokyo, Japan).

### Confocal immunofluorescence microscopy

After transfection, the target cells were fixed with a mixture of equal volumes of methyl alcohol and acetone for 15 min, washed three times with PBS, and blocked with PBS containing 10% bovine serum albumin (BSA) for 1 h at room temperature. Then, the cells were incubated with the primary antibodies overnight at 4 °C, followed by incubation with secondary antibodies (ProteinTech Group) for 1 h. Samples were washed five times with PBS containing 0.01% Tween 20 and 1% BSA. Mounting was performed with Vectashield mounting medium with DAPI (Vector Laboratories), and the cells were visualized by confocal laser microscopy (FLUOVIEW FV1000; Olympus, Tokyo, Japan).

### Nuclear extraction

The cell samples were collected and washed in cold PBS. The cells were resuspended in buffer A (10 mM Tris-HCl [pH 7.4], 5 mM MgCl_2_, 10 mM NaCl, 1 mM DTT, 10% protease inhibitor mixture) for 15 min on ice before 0.5% NP-40 in buffer A was added, and the mixture was vortex-mixed for 10 s. Nuclei were pelleted by centrifugation at 15,000 g for 1 min, and cytosolic–protein-containing supernatants were collected. After the pellet was washed in buffer A, it was resuspended in buffer C (20 mM HEPES-KOH [pH 7.9], 1.5 mM MgCl_2_, 0.5 M NaCl, 1 mM DTT, 0.2 mM EDTA, 1% NP-40, 10% protease mixture inhibitor), vortex-mixed for 15 s, and incubated on ice for 10 min; this process was repeated three times. After centrifugation at 15,000 g for 30 min, nuclear–protein-containing supernatants were collected. Proteins were detected by western blot analysis.

Real-time PCR (qPCR) analysis, Transfection and luciferase reporter assays, VSV plaque assays and MTS cell viability assay can be found in supplementary information and Supplementary Table 1.

### Statistical Analysis

All experiments were reproducible and were carried out in triplicate or quadruplicate. Each set of experiments was repeated at least three times with similar results, and a representative one is shown. The results are presented at the means ± s.d. Student’s t test for paired samples was used to determine statistical significance. Differences were considered statistically significant at a value of P < 0.05 was considered statistically significant.

## Additional Information

**How to cite this article**: Cao, Z. *et al.* Methylcrotonoyl-CoA carboxylase 1 potentiates RLR-induced NF-κB signaling by targeting MAVS complex. *Sci. Rep.* **6**, 33557; doi: 10.1038/srep33557 (2016).

## Supplementary Material

Supplementary Information
