# Supplementary material for: Methylcrotonoyl-CoA carboxylase 1 potentiates RLR-induced NF-κB signaling by targeting MAVS complex
Source: Sci Rep. 2016 Sep 15;6:33557. doi: 10.1038/srep33557 (PMC5024325; doi:10.1038/srep33557)
Supplement: Supplementary Information [file srep33557-s1.pdf]

Methylcrotonoyl-CoA carboxylase 1 potentiates RLR-induced NF-κB signaling by targeting MAVS complex

Zhongying Cao; Zhangchuan Xia; Yaqin Zhou; Xiaodan Yang; Hua Hao; Nanfang Peng; Shi Liu; Ying Zhu*

Real-time PCR (qPCR) analysis

Total RNA was isolated with TRIzol (Invitrogen) and extracted according to the manufacturer's protocol. Quantitative PCR assays were performed using the ABI StepOne Real-Time PCR system (Applied Biosystems, Waltham, MA) and SYBR RT-PCR kits (Applied Biosystems). All data represent relative expression of target gene to that of reference gene GAPDH with efficiency correction.

Supplementary Table 1. The primers used for qPCR in this study.

gene	5'primer (5' to 3')	3'primer (5' to 3')
GAPDH	AAGGCTGTGGGCAAGG	TGGAGGAGTGGGTGTCG
MCCC1	ACAACAGCCACAGGAAGAAACATTA	TTGAATGATTTTCTCCATAGATAGG
IFN-α	TTTCTCCTGCCTGAAGGACAG	GCTCATGATTTCTGCTCTGACA
IFN-β	AAAGAAGCAGCAATTTTCAGC	CCTTGGCCTTCAGGTAATGCA
IFN-λ1	CTTCCAAGCCCACCCCAACT	GGCCTCCAGGACCTTCAGC
Mx1	GCCGGCTGTGGATATGCTA	TTTATCGAAACATCTGTGAAAGCAA

PKR	AGAGTAACCGTTGGTGACATAACCT	GCAGCCTCTGCAGCTCTATGTT
IL-6	GGTACATCCTCGACGGCATCTCA	TGCACAGCTCTGGCTTGTTCCTC
IL-8	GGTGCAGTTTTGCCAAGGAG	TTCCTTGGGGTCCAGACAGA
IL-1β	CAGAAGTACCTGAGCTCGCC	CATGGCCACAACAACTGACG
TNFα	CTTCTCGAACCCCGAGTGAC	ATGAGGTACAGGCCCTCTGA

Transient Transfection and Luciferase Reporter Gene Assays

Cells were plated in 24-well plates (1×10^5 cells/well) and grown to 80% confluence at the time of transfection. Cells were transfected with reporter plasmids together with specific expression vectors or shRNA expression vectors using Lipofectamine 2000 (Invitrogen). Vector pRL-TK (Promega, Madison, WI) was used as an internal control to calculate the transfection efficiency. Cells were serum-starved for another 24 h before being harvested for luciferase activity assays. The assay results are expressed as relative luciferase activity.

VSV plaque assays

The target cells were grown in 12-well plates and transfected with plasmid. Twenty-four hours after transfection, cells were infected with VSV (MOI = 1). After 1 h, cells were washed with phosphate-buffered saline (PBS), and fresh medium was added. After 24 h, the supernatants were harvested, diluted to 10^{-6}, 10^{-5}, 10^{-4}, 10^{-3}, 10^{-2}, and 10^{-1} and used to infect confluent Vero cells cultured on 24-well plates. At 1 h post-infection,

supernatants were removed and Vero cells were washed once with PBS, and then overlaid with a mixture of warm 3% low–melting-point agarose and fresh medium. At 72 h post-infection, cells were stained with 0.2% crystal violet for 2 h, and then the overlay was removed. Plaques were counted, averaged, and multiplied by the dilution factor to determine the viral titer (PFU/mL).

MTS Cell Viability Assay

A549 cells were seeded into 96-well plates at 5000 cells/well. After adhesion, cells were transfected with control siRNA or siRNA targeting MCCC1 using Lipofectamine 2000 (Invitrogen). Forty-eight hours after transfection, MTS assays were performed using the MTS cell viability kit (Promega) according to the manufacturer’s instructions. Cell viability was then calculated.

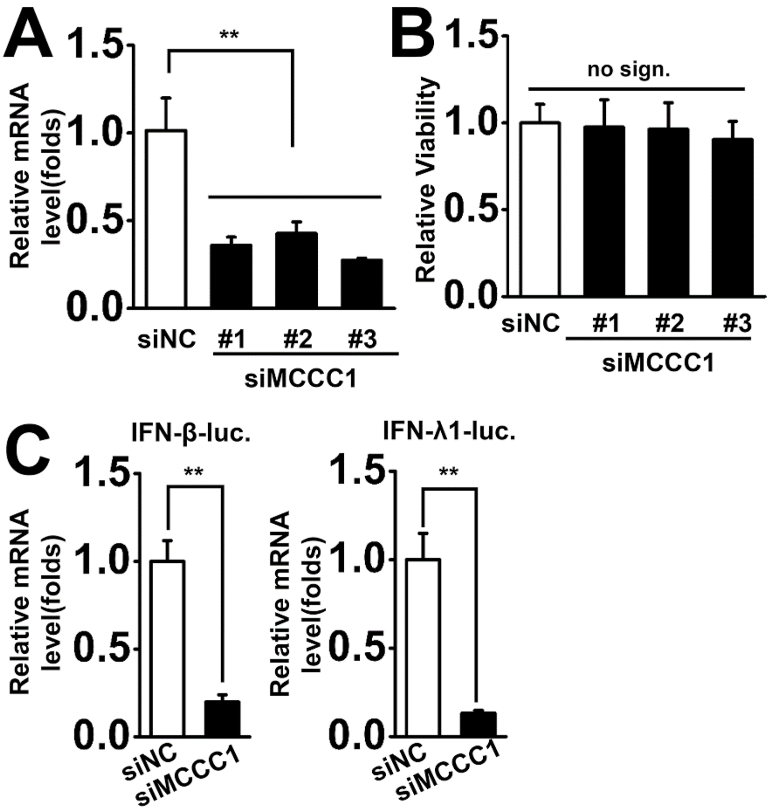

Supplementary Figure S1. (A) A549 cells were transfected with different MCCC1-specific siRNA or nonsense control siRAN for 48h. Then the total RNA was isolated and the mRNA level of MCCC1 was detected by qPCR. **$p < 0.01$ (one-way ANOVA). (B) A549 cells were transfected with different MCCC1-specific siRNA and nonsense control siRNA. Fourty-eight hours after transfection, MTS assays were performed using a MTS cell viability kit (Promega) according to the manufacturer's instructions. Cell viability was then calculated. no sign., no significant difference. (C) qPCR analysis of A549 cells co-transfected with siRNAs targeting MCCC1 or nonsense control siRNA and IFN-β or IFN-λ1 luciferase reporter plasmids together with pRL-TK (control) for 24 h, followed by infection with SeV for 12 h. **$p < 0.01$ (one-way ANOVA). All experiments were repeated at least three times with consistent results.
